# Introduction of managed entry agreements in Korea: Problem, policy, and politics

**DOI:** 10.3389/fphar.2023.999220

**Published:** 2023-04-13

**Authors:** Hyungmin Kim, Brian Godman, Hye-Young Kwon, Song Hee Hong

**Affiliations:** ^1^ College of Pharmacy, Seoul National University, Seoul, Republic of Korea; ^2^ National Health Insurance Service, Wonju, Republic of Korea; ^3^ Division of Pharmacoepidemiology, Strathclyde Institute of Pharmacy and Biomedical Sciences, University of Strathclyde, Glasgow, United Kingdom; ^4^ Division of Public Health Pharmacy and Management, School of Pharmacy, Sefako Makgatho Health Sciences University, Pretoria, South Africa; ^5^ School of Pharmaceutical Sciences, Universiti Sains Malaysia, Penang, Malaysia; ^6^ Division of Biology and Public Health, Mokwon University, Daejeon, Republic of Korea; ^7^ Research Institute of Pharmaceutical Sciences, Seoul National University, Seoul, Republic of Korea

**Keywords:** managed entry agreement (MEA), risk sharing agreement (RSA), confidential discount, finance and outcome based contract, kingdon’s model

## Abstract

**Objectives:** This study aimed to understand Managed Entry Agreements (MEAs) in Korea through the framework of three streams of the policy window model and its practical management and impact on pricing and reimbursement scheme.

**Methods:** An extensive literature review based on Kingdon’s model was conducted. We also performed descriptive analyses of MEA implementation using data on medicines listed in Korea and compared its MEA scheme with four different countries.

**Results:** As per problem streams, patients with rare disease or cancers have considerable difficulties in affording their medicines and this has challenged the drug benefit system and raised an issue of patient’s access. Policy streams highlighted that MEAs were introduced as a benefit enhancement plan for four major diseases since January 2014. MEAs have also been strengthened as a bypass mechanism to expand the insurance coverage especially for new premium-priced medicines under Moon Care (Listing all non-listed services). In descriptive analysis of MEAs, a total of 48 medicines were contracted as MEAs from January 2014 to December 2020, accounting for 73.4% of listed medicines for cancer or rare diseases and 97.9% of the cases were finance-based contracts. Meanwhile, outcome-based contracts such as CED accounted for only 2.1%. The application of MEAs differs across countries, resulting in a kappa coefficient of 0.00–0.14 (United Kingdom 0.03, Italy 0.00, Australia 0.14), indicating a lack of consistency compared to South Korea.

**Conclusion:** MEAs, which were introduced as a bypass mechanism, have now superseded the standard process for anticancer agents or orphan drugs. Further studies are needed to evaluate the impact of the confidential agreements and effectiveness of new high-priced medicines with limited clinical data at launch.

## Introduction

Global expenditure on medicines has been rising at a compounded rate of 3%–6% per annum in recent years, enhanced by growing sales of new premium priced biological medicines, to treat patients with complex diseases including cancer and orphan diseases (IQVIA, 2019). The funding of new high-priced medicines for these disease areas is difficult to sustain, especially in countries that seek to attain or retain universal access. This alongside funding increased medicine volumes with aging populations and changes in clinical practice to treat diseases more aggressively (Godman et al., 2018; Godman et al., 2021). For instance, it has been estimated that expenditures on new oncology medicines approved in the United States in 2018 alone, could be as high as US$39.5 billion per year, if these were prescribed to all eligible patients (DeMartino et al., 2021). These challenges have resulted in multiple measures across countries to re-evaluate pricing and reimbursement considerations for new medicines (Godman et al., 2018; Godman et al., 2021). Alongside this, there have been ongoing reforms to release savings by increasing the use of low-cost multiple sourced medicines and biosimilars, without compromising care (Godman et al., 2021; Moorkens et al., 2021; Godman et al., 2022).

Funding new medicines for oncology and orphan diseases has become an increasing challenge with rising prices and limited health gain for a number of new medicines, driven by the emotive nature of these disease areas (Haycox, 2016; Cohen, 2017; Luzzatto et al., 2018; Hollis, 2019; Godman et al., 2021). However, this is not always the case (Molto et al., 2020). Alongside this, we are seeing new medicines for these disease areas often being launched with limited data and considerable uncertainty, which can be an issue to health authorities with finite resources and many competing demands when expectations are not met (FDA, 2018; IQVIA Institute for Human Data Science, 2018; Pontes et al., 2020). However, this has to be balanced against the unmet need for new medicines for cancer and orphan diseases, to reduce projected morbidity and mortality (Orphanet Report Series, 2021; Sung et al., 2021). In view of these challenges, health authorities and their advisers across countries have been evaluating potential ways to move forward with pricing and reimbursement of new premium-priced medicines, especially for oncology and orphan diseases, where most new medicines are being developed (Lasalvia et al., 2019; Lee et al., 2019; Godman et al., 2021; IQVIA, 2022). These deliberations have been accelerated by the launch of new advanced therapy medicinal products (ATMPs), including new high-priced gene therapies (Barlow et al., 2019; Shukla et al., 2019).

Proposed approaches among health authorities and their advisers to deal with these challenges include establishing minimum effectiveness criteria (oncology medicines), MEAs, multi-criteria decision analyses (MCDAs), multi-indication pricing (including for oncology medicines across tumours and stages) and transparent pricing models (Wild et al., 2016; Uyl-de Groot and Lowenberg, 2018; Hsu et al., 2019; Lasalvia et al., 2019; Moon et al., 2020; Godman et al., 2021). Overall, MEAs, also called risk sharing arrangements, have been increasingly seen and used among payers across countries to facilitate access to new premium priced medicines (Adamski et al., 2010; Ferrario and Kanavos, 2015; Ferrario et al., 2017; Nazareth et al., 2017; Pauwels et al., 2017; Bouvy et al., 2018; OECD, 2020; Zampirolli Dias et al., 2020). The purpose of the MEAs varies across countries. However, common rationale includes the need to facilitate access to new medicines, particularly for cancers or rare diseases in which uncertainty of the effectiveness and the finance/budget is inherently embedded alongside potential concerns with requested prices (Morel et al., 2013; Kanavos et al., 2017; Godman et al., 2021).

Since December 2013, the Korean government enacted a regulation permitting MEAs within the National Health Insurance scheme for new high-priced medicines. Starting with clofarabine in December 2013, the introduction of MEAs has been accelerating. Recently, the Korean government planned to enhance the application of MEAs in order to facilitate access to high priced medicines (Ministry of Health and Welfare MOHW, 2017). This built on previous studies that had investigated the positive impact on patient care with increased access to new treatments, particularly those for cancer and orphan diseases (Kim et al., 2017; Yoo et al., 2019; Kim et al., 2020; Lee, 2021). Yoo et al. (2019) reported that the introduction of MEAs and cost-effectiveness analysis waiver track, alongside increasing patient’s co-payments where there were concerns with their cost-effectiveness, contributed to improving patient access to new treatments. Kim et al. (2017) also investigated the positive impact of MEAs on access to new anti-cancer medicines and on increasing their likelihood of being listed within the Korean pharmaceutical benefit scheme.

Such a policy introduction can be explained through Kingdon’s traditional theory. The policy window model describes how a policy is set on an agenda and develops into a policy (Kingdon; Kingdon, 1995; Gilla et al., 2017). According to their model, agenda setting is defined as three process streams flowing through the system—streams of problems, policies, and politics. At some critical junctures, the three streams are joined while they develop independently through their own dynamics and rules, with the greatest policy changes growing out of the coupling of problems, policies, and solutions. The result of the convergence of the three streams is the opening of a “policy window,” which allows advocates of a particular issue to place these on the policy agenda (Kingdon, 1995).

Consequently, this study aimed to illuminate MEAs in South Korea through the framework of the three streams of the policy window model, in order to understand the context in which MEAs were introduced and developed as a pharmaceutical policy in South Korea. This has not been addressed in previous studies. In addition, this study also provides empirical analysis for MEAs within an international comparative approach to guide further research.

## Materials and methods

Based on Kingdon’s model (Kingdon; Kingdon, 1995; Gilla et al., 2017), we first investigated problems of access to medicines alongside subsequent policies and political changes based on an extensive review of the existent secondary literature. A search was carried out by using multiple keywords in the following database: PubMed, Web of Science, and Google Scholar. The keywords included “risk sharing,” “managed entry agreement,” “coverage with evidence development,” “expenditure cap,” “volume cap,” “confidential discount,” “conditional treatment continuation,” “patient access scheme,” “performance- or outcome-based,” “budget- or finance-based” in both Korean and English. The search yielded published papers and relevant documents, government policy reports, regulations, and press releases.

In addition, descriptive analyses of MEA implementation were conducted by using data on medicines listed in Korea. As of December 2020, medicines listed under the MEAs were extracted from the National Health Insurance (NHI) drug reimbursement list pertaining to active pharmaceutical ingredients, strengths, brand names, manufacturers, maximum reimbursement prices, listing dates, designated orphan drugs and subject of MEA contracts, notified by the MOHW on a monthly basis. The list is available from the public website (www.hira.or.kr) (Health Insurance Review & Assessment Service HIRA, 2022a; Health Insurance Review & Assessment Service HIRA, 2022b). A total of 48 medicines have been contracted with MEAs since 2014, six of which have already expired (See Supplementary Appendix). Subsequently, these medicines were analysed in accordance with their indications, types of MEAs, and price changes after contract termination, and compared to those in Australia, Italy, and the United Kingdom. Data of medicines under MEAs in each country were sourced from official information notified by public agencies [NICE (National Institute for Health and Care Excellence), 2022; AIFA (Agenzia Italiana del Farmaco); PBS (Pharmaceutical Benefits Scheme), 2022]. Furthermore, an agreement test (McHugh, 2012) was conducted to determine whether the MEA was applied consistently across countries.

## Results

### Problems: Access to medicines

#### Refusal to supply ultra-orphan medicines

In 2001, Korea experienced a refusal by Novartis to supply Glivec^®^ (Imatinib) for leukaemia treatment at a suggested price from MOHW. Subsequently, this strategy has often been employed by multi-national pharmaceutical companies, working together with patient’s advocacy groups to put pressure on governments to accept the higher prices of medicines, in view of maintaining global reference pricing goals. Examples of the strategy to raise price of medicine were found in the literature (Kwon and Yang, 2010). Starting with Glivec^®^ (Imatinib) in 2001 and up till 2010, seven cases of refusal by multi-national drug companies to launch their medicines at lower prices were found. Kwon and Yang (2010) summarized these cases as follows: Imatinib (Glivec^®^)- Leukemia, 2001; Enfuvirtide (Fuzeon^®^)- HIV/AIDS, 2004; Darunavir (Frezista^®^)- HIV/AIDS, 2008; Galsulfase (Naglazyme^®^)- Mucopolysaccharidosis IV, 2009; Idursulfase (Elaprase^®^)^_^ Mucopolysaccharidosis II, 2009; Alglucosidase alfa (Myozyme^®^)- Pompe disease, 2009; and Eptacog alfa (Novoseven^®^)- Hemophilia, 2010. They concluded that the refusal by MOHW to fund new medicines at high prices had four common characteristics: Firstly, the medicines targeted rare or life-threatening diseases with no substitutable medicines. Secondly, the suppliers were multi-national pharmaceutical companies with a monopolistic position. Thirdly, pharmaceutical companies refused to provide a supply of medicines, due to their dissatisfaction with the prices set by Korean government. Lastly, the companies were operating a patient support program, providing free of charge medicines after a drug supply was refused (Kwon and Yang, 2010).

Recently, a case of iodised fatty acid (Lipidol^®^) was added. In 2018, Guerbet Ltd., a French manufacturer, decided not to supply this medicine to Korea without raising its price by 500% (Lee, 2018). In response, the National Health Insurance Service (NHIS) increased the price by 360% and agreed on the supply obligation of the company, as well as the measures to adopted when the supply was stopped. This was also consistent with the common characteristics in the previous cases. In this particular situation in Korea, MEAs were considered a method to set visible and effective prices that were different from the requested list prices, and have been considered an alternative to addressing the refusals of companies to launch new medicines at the prices suggested by MOHW (Health Insurance Review & Assessment Service HIRA, 2022b).

#### Extra-billing, financial burden to patients

Although South Korea achieved universal healthcare in 1989 (Yu and Anderson, 1992), and the public share in pharmaceutical expenditures is as high as the Organisation for Economic Co-operation and Development (OECD) average, and higher than the United States and Canada, where the public share is less than 40% (Jeong and Shin, 2013), it has long been common practice among doctors to prescribe medicines not covered by the NHI. Since 2007, the introduction of the positive list system, the clinical and economic value of the applied medicines—in other words, its cost effectiveness—is crucially taken into consideration while making reimbursement decisions (Bae et al., 2016; Kwon and Godman, 2017). Consequently, new premium-priced medicines that fail to demonstrate their cost-effectiveness are determined to be non-reimbursable. This has continuously caused an access issue for pertinent new medicines, due to the financial burden to patients. Typically, new premium priced medicines, i.e., those for cancer or an orphan disease that are frequently prescribed in clinical settings, but not considered cost effectiveness cause financial crisis for patients. Therefore, Cancer patients or patients with rare diseases have considerable difficulties purchasing these medicines, and this has challenged the drug benefit system and raised the issue of patients’ access to these medicines, especially when recommended for patient management.

### Policy streams for MEAs

#### Solution for ultra-orphan drug supply refusal: Refund scheme as a pilot plan

Since 2009, MEAs have been suggested by multi-national pharmaceutical companies as a potential way to facilitate patients’ access to ultra-orphan medicines in Korea. The “Refund scheme,” a confidential discount defined by Wenzl and Chapman (2019) and categorized as a financial-based MEA (Bouvy et al., 2018), was implemented in 2009. This plan was the first to set dual prices for particular medicines: a listing price open to the public, and an actual price always lower than the listing price but confidential to the public. Under this scheme, the supplier should pay back the difference in costs between the list price and the actual price to the NHIS. Given that patient’s co-payments are primarily calculated as a proportion of the total drug costs, i.e., 30% of total drug costs for pharmacy services, the new medicines eligible for this plan were limited to those that are included in the rare and debilitating disease support program (RDSP), fully funded by the government. This is because the co-payments for these high-cost medicines were exempted by the government subsidies and no additional co-payment costs, based on the difference in the dual prices, could be charged. Three medicines, including Naglazyme^®^ (Galsulfase) for Mucopolysaccharidosis Type IV in 2009, Myozyme^®^ (Alglucosidase alfa) for Pompe disease in 2009 and Soliris^®^ (Eculizumab) for Paroxysmal nocturnal hemoglobinuria in 2012 were contracted under the pilot Refund scheme (Ministry of Health and Welfare in Korea MOHW, 2012). Since then, the demand for listing new premium priced anti-cancer medicines, as well as the request to extend this Refund scheme to other drugs not limited to the RDSP have continued, and this pilot scheme is currently being operated as one type of MEA (Ministry of Health and Welfare in Korea MOHW, 2013a; Ministry of Health and Welfare in Korea MOHW, 2013b).

#### Benefit enhancement plan (BEP) for four major diseases

With the inauguration of the former government in 2012, there have been substantial changes in health policies under the framework of expanding the benefit of the NHI targeting four major diseases, including cardiovascular, cerebrovascular, orphan diseases, and cancers in June 2013 (Ministry of Health and Welfare MOHW, 2013), which was an election pledge of the former president Park. The four major diseases were a group of diseases with the largest increase in medical costs and out-of-pocket expenses in recent years. MEAs were introduced as a bypass mechanism to expand insurance coverage in these four major diseases from January 2014.

The Ministry of Health and Welfare (MOHW) (Ministry of Health and Welfare in Korea MOHW, 2013b) explained the need for MEAs as follows: “*Since the Korean National health Insurance has been running a positive system for listing cost-effective drugs, it has been difficult to provide reimbursement when high-priced new drugs cannot demonstrate its cost-effectiveness. The non-reimbursed high-cost medicines were identified as the main cause of increasing financial burden to patients. As a result, the listing system needs to be revised to improve patient accessibility to the treatments without compromising the principal of the positive listing system*.”

The former government declared that high-cost medicines treating these four major diseases that were financially burdensome to patients could be subsidized through MEAs. As a result, MEAs played a role to help the cost of high-priced cancer drugs and orphan drugs be reimbursed. Accordingly, high-cost anti-cancer drugs, including Cetuximab (authorized in 2009) and Renalidomide (authorized in 2009) that had long been classified as non-reimbursable, were added to the drug reimbursement list under the Refund scheme in 2014 (Health Insurance Review & Assessment Service HIRA, 2022a).

### Politics streams

#### Moon Care: Listing all non-listed services

With the inauguration of the Moon Jae-In government in 2017, the BEP of the former regime was modified and expanded to all conditions, not limited to four major diseases. A core principle of Moon Care was to eliminate non-reimbursable services by “listing all non-listed services” (Park, 2017; Kang, 2018). The government was confident that the price and use of the non-listed services could be controlled by doing so (Kim, 2014).

Under the Moon Care, MEAs have been strengthened as a bypass mechanism to expand the insurance coverage, especially for new premium priced medicines. As a result, as of July 2019, a total of 421 non-reimbursable medicines were listed for reimbursement. In addition to listing previously unlisted medicines due to concerns with their cost-effectiveness, the government listed medicines with earlier concerns about their cost effectiveness and has expanded the scope of reimbursement for listed medicines, including medicines contracted under the MEA (Ministry of Health and Welfare MOHW, 2019). Moreover, the relaxation of previous measures has made more medicines eligible for MEAs and extended the duration of MEA contracts (Ministry of Health and Welfare, 2020). Consequently, new medicines, including the late-competitive products of the MEA-applied drugs, medicines applied to CEA exemptions, and those with conditional approval (i.e., without phase III trials) have also become eligible for MEAs (Ministry of Health and Welfare, 2020).

**FIGURE 1:**
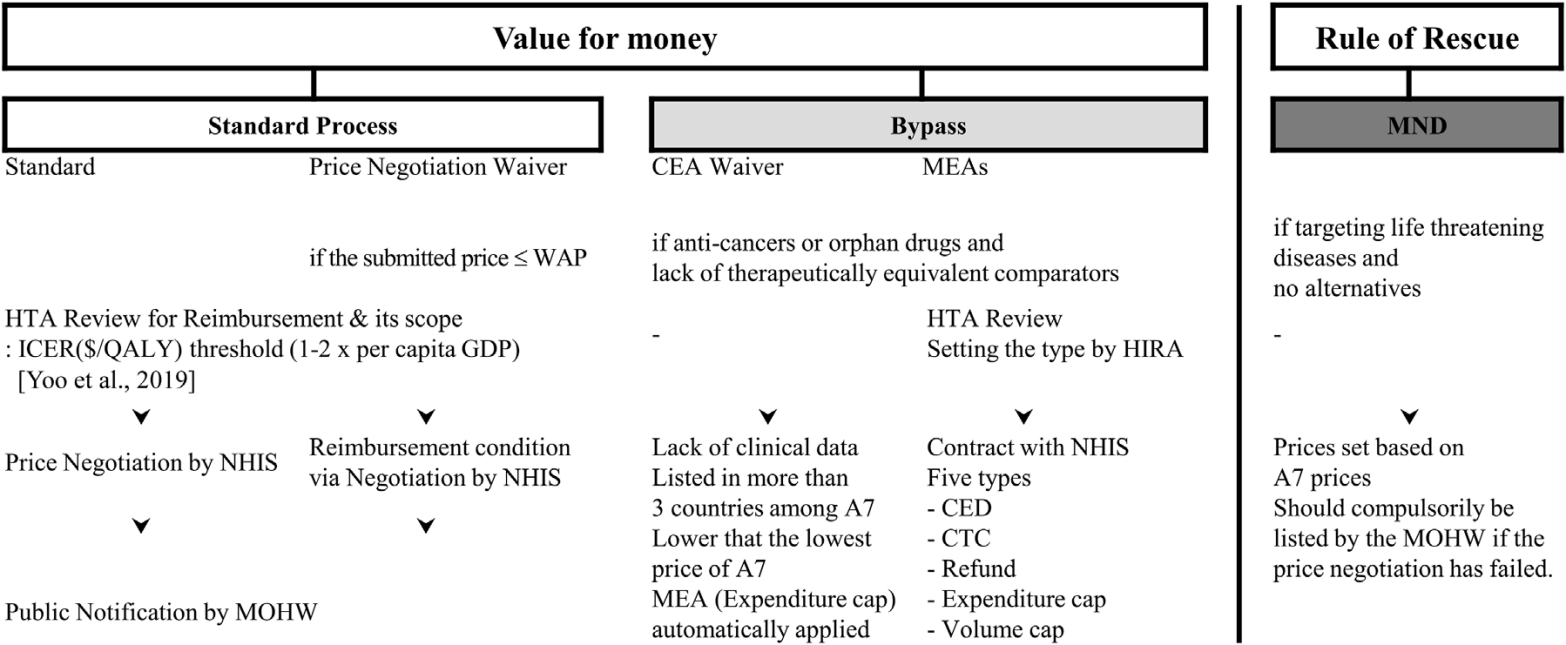
General scheme for Drug Reimbursement in Korea. Abbreviations: MND, medically necessary drugs; CEA, cost-effectiveness appraisal; MEA, managed entry agreement; WAP, weighted average price of comparators; HTA, health technology assessment; ICER, incremental cost-effectiveness ratio; QALY, quality adjusted life years; GDP, gross domestic production; HIRA, National Health Insurance Review and Assessment Agency; NHIS, National Health Insurance Service; MOHW, Ministry of Health and Welfare; CED, coverage with evidence development; CTC, conditional treatment continuation; A7, Seven advanced countries including United States, United Kingdom, Switzerland, Italy, France, Germany, and Japan.

Together with MEAs, other interventions were also introduced to bypass the current pharmaceutical benefit policies (Figure 1). For example, exemptions of cost-effective analysis, exemptions of price negotiations, and a flexible application of ICER (Incremental cost-effectiveness ratio) thresholds for cancer or rare disease drugs (Ministry of Health and Welfare MOHW, 2014). According to the principle of the BEP, new high-cost medicines with concerns about their cost-effectiveness could be listed under certain criteria, which may be in conflict with the positive list system (PLS).

### Administration of MEAs

#### Types of MEAs

**FIGURE 2:**
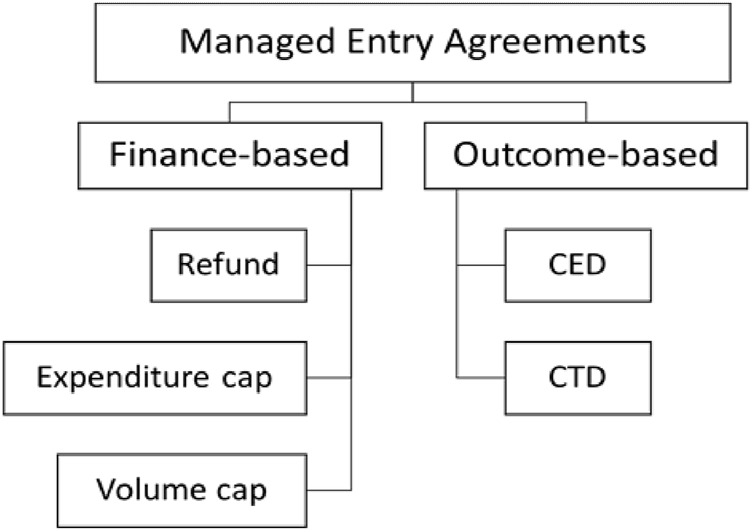
Five types of MEAs in South Korea. Note: Refund corresponding to the confidential discount. CED, Coverage with Evidence Development; CTD, Conditional treatment continuation.

Basically, five types of MEAs were specified by regulations (Ministry of Health and Welfare, 2013): expenditure cap, volume cap (utilization cap per patient), refund (a confidential discount, in other words, double pricing), coverage with evidence development (CED) and conditional treatment continuation (CTC or money back guarantee) (Figure 2).

However, combinations of these types of MEAs are also acceptable when suggested by manufacturers. The definitions for each type are formulated as below, with manufacturers obliged to pay back $E_{∆}$ to the NHIS.

⁃ Expenditure cap: $E_{∆}=E_{actual}-E_{capped}=P*Q-E_{capped}$ , if $E_{acutal}>E_{capped}$

⁃ Utilization cap per patient (volume cap): $E_{∆}=P*\left(Q_{actual}-Q_{capped}\right)$ , if $Q_{actual}>Q_{capped}$

⁃ Refund: $E_{∆}=\left(P_{listed}-P_{actual}\right)*Q_{actual}$

⁃ Conditional treatment continuation: $E_{∆}=P*Q$ , in case of no response
⁃ Coverage with Evidence Development: $E_{∆}=E_{total}*R$ , R is achievement rate (%) compared to the contracted target performance

The expenditure cap is a structure where the total cost of new medicines is fixed and the expenditures above the fixed cost ( $E_{∆}$ ) should be paid back to the NHIS. The volume cap is a structure in which the total quantity of the new medicine is fixed, and the total amount can be obtained by multiplying the difference between the capped volume and the real one by the price. In this scheme, the utilized quantity of the medicine would be variable and key to determining the repayable cost ( $E_{∆}$ ) that will be returned to the NHIS. Both schemes would be effective if either the total cost or the total quantity used should exceed the contractually fixed value. The Refund scheme aims to set the return cost ( $E_{∆}$ based on the difference between two prices, the listing and actual price, by multiplying the quantity prescribed. In these schemes, the quantity utilized would be uncertain and key to determining the repayable cost ( $E_{∆}$ ). Unlike these abovementioned schemes, which belong to the finance-based MEAs (Bouvy et al., 2018), CTC and CED are classified as outcome-based schemes (Adamski et al., 2010). The CTC is basically to cover the costs of a new medicine when an effective response is proved, while the CED provides temporal funding coverage for a new medicine during the evaluation of its performance in routine clinical care. Depending on the study results, coverage may be maintained, withdrawn, or extended, or prices may be adjusted (Wenzl and Chapman, 2019; Dabbous et al., 2020).

#### Subjects eligible to MEAs

To be subject to MEAs, the following criteria need to be satisfied: medicines for rare diseases or cancers that have no alternatives, or no therapeutically equivalent medicines or treatments that can be used for life threatening diseases. Alongside this, when the new medicines are recognized as being necessary in consideration of the disease severity, their social impact, and other healthcare impacts (Ministry of Health and Welfare, 2013).

A total of 48 medicines contracted as MEAs were collected from the monthly notification of the HIRA’s website, from January 2014 to December 2020 (see Supplementary Appendix).

As seen in Table 1, all but one medicine contracted with the MEAs was approved by the Ministry of Food and Drug Safety for use in cancers and rare diseases. Out of these, Dupilumab (Dupixent^®^), targeting atopic dermatitis, was listed *via* the MEAs when considering the severity of its indicative conditions. Now, six drugs have had their MEA contracts terminated. Renalidomide (Revelimid^®^) and Pirfenidone (Pirespa^®^) have automatically expired, since the generic versions were available (Choi, 2017a; Choi, 2017b). Crizotinib (Xalkori^®^) has had its contract terminated due to the listing of its competitor; Galsulfase (Naglazyme^®^) and Eculizumab (Soliris^®^) have expired because the suppliers wanted to terminate the contract. The contract that Clofarabine (Evoltra^®^) had with the CED ended after it had demonstrated its effectiveness. Consequently, its listing status and price did not change after the contract expired.

**TABLE 1:** Indications of MEAs.

ATC category		No. of drugs (%)	Rare diseases or cancer
A	Alimentary tract and metabolism	4 (8.3%)	Rare
B	Blood and blood forming organs	2 (4.2%)	Rare
D	Dermatologicals	1 (2.1%)	N/A
L	Antineoplastic and immunomodulating agents	38 (79.2%)	Cancer, and/or rare
M	Musculo-skeletal system	1 (2.1%)	Rare
N	Nervous system	1 (2.1%)	Rare
V	Various	1 (2.1%)	Rare
Total		48 (100%)	

#### Impacts of MEAs

**FIGURE 3:**
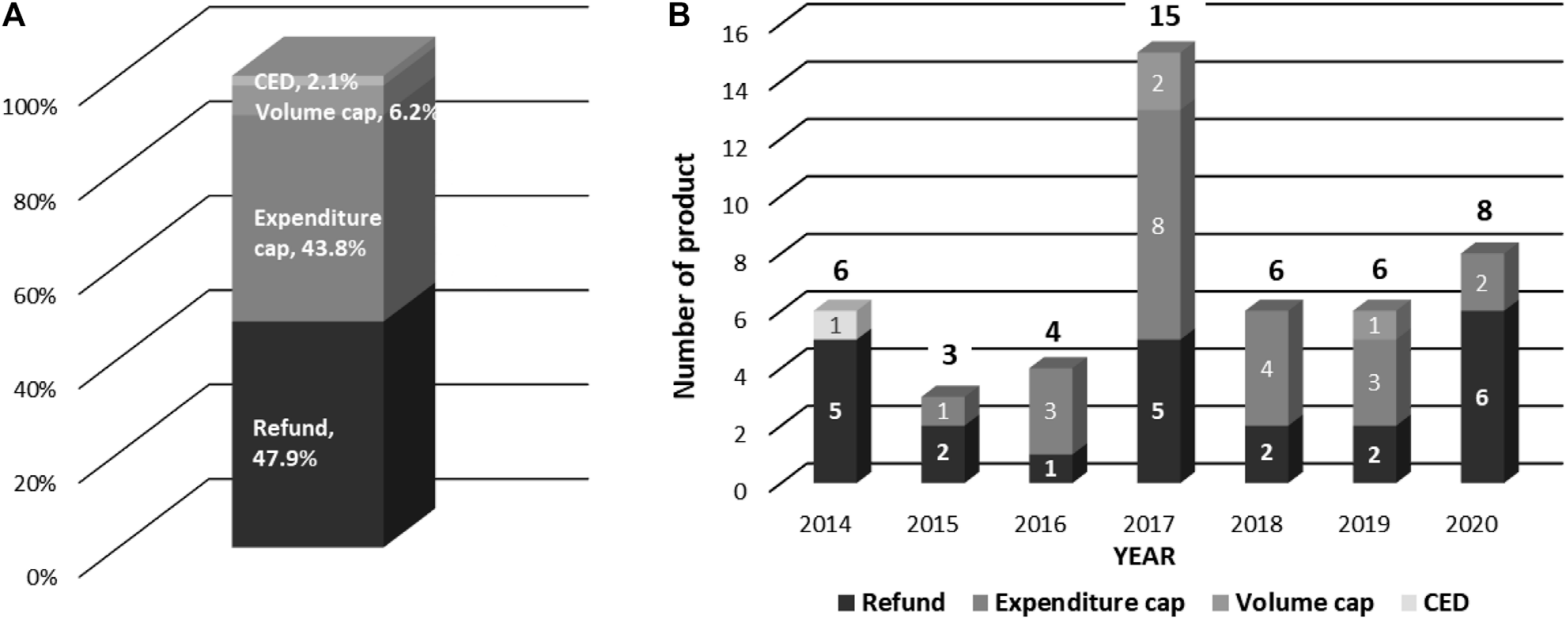
Types of MEAs and Number of products by year. **(A)** Types of MEAs **(B)** Yearly Number of Products contracted with MEA.

Current applied types of MEAs are depicted in Figure 3A. Out of these cases, 97.9% were finance-based contracts, such as refunds, expenditure caps and volume caps. Meanwhile, outcome-based contracts such as CED accounted for only 2.1%. The most prevalent type of MEAs in Korea was found to be under the Refund scheme, i.e., a dual pricing scheme (47.9%). As shown in Figure 3B, MEAs have been actively applied in 2017, compared to other years (31.3%). Overall, an increasing trend of MEA cases over the years has been observed (Figure 3).

**FIGURE 4:**
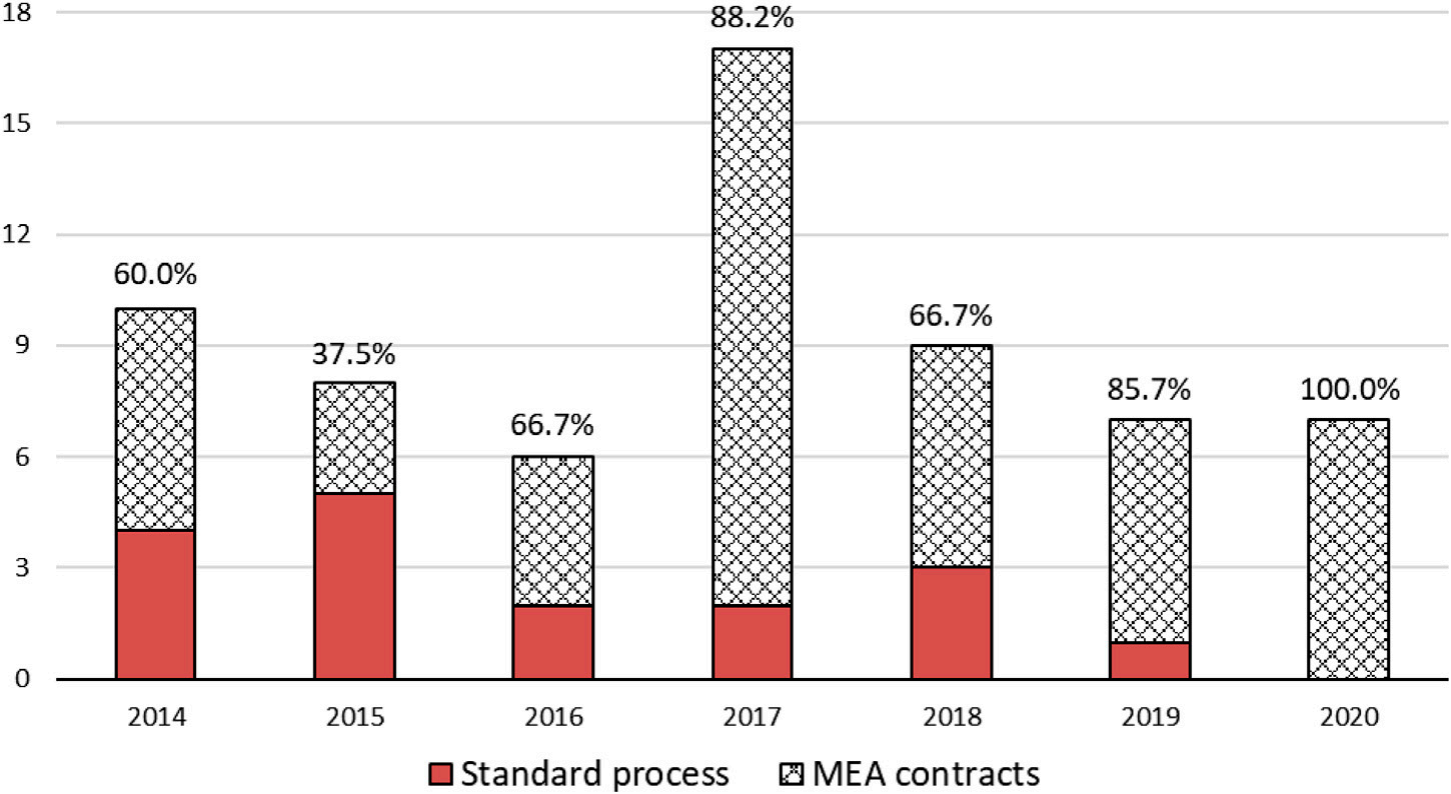
Number of listed medicines for cancers or rare disease by year (2014–2020).

Since its implementation in 2014, the MEAs have become a common route for new anticancer medicines or those for orphan diseases. As shown in Figure 4, a total of 64 new medicines for cancer or rare diseases were requested to be listed from 2014 to 2020. Of these, 73.4% (47 drugs) were listed *via* MEAs. As the years passed, the exceptional route became the standard one.

**FIGURE 5:**
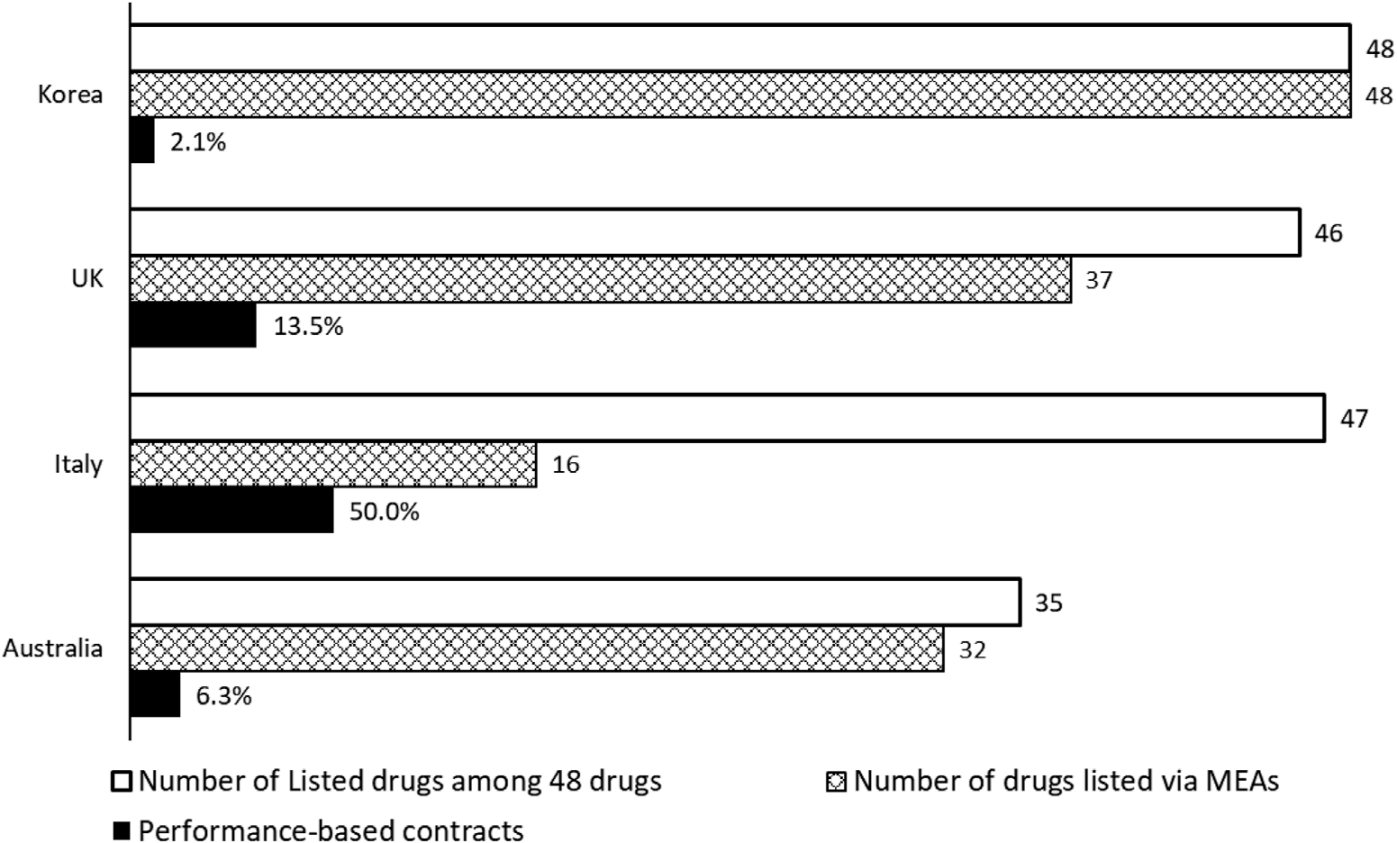
Comparison with the United Kingdom, Italy, and Australia for the 48 drugs.

Forty-eight medicines contracted with MEAs in South Korea were analysed in light of their listing status and MEA applications in Australia, Italy, and the United Kingdom [NICE (National Institute for Health and Care Excellence), 2022; AIFA (Agenzia Italiana del Farmaco); PBS (Pharmaceutical Benefits Scheme), 2022]. Most of the 48 new medicines under consideration were available within the National Health System in the United Kingdom and Italy, while only 72.9% were covered by the Australian Pharmaceutical Benefit System (Figure 5). In the United Kingdom and Australia, 77.1% and 66.7% of these 48 new medicines were listed under the MEA contracts, respectively, compared to only 33.3% in Italy. Among the medicines listed *via* MEAs, Korea showed the lowest number (2.1%, 1 out of 48) of outcomes-based contracts, while Italy showed the most at 50.0% (8 out of 16). The comparative analysis showed that the application of MEAs differs across countries, resulting in a kappa coefficient of 0.00–0.14 (United Kingdom 0.03, Italy 0.00, Australia 0.14), indicating a lack of consistency compared to Korea (McHugh, 2012).

Time consumed for listing of these medicines was shortened from an average of 698 days in 2014 to 386 days in 2020. Overall, it has taken an average of 516 days (median: 357, min: 196, max; 1,685) from submission to listing for the medicines contracted with MEAs.

Changes in listing prices during and after MEA contract expiry for six drugs were analysed. As shown in Table 2, the average contract period was 5.4 years, and the price was set 1.6 times higher during the contract period.

**TABLE 2:** Listing Price Changes During and After the MEA contracts. Unit: USD, Years.

Active ingredient	Type of MEAs	Listing price per unit		Contract period	Estimated refund rate [(A-B)/A]
		Under MEAs [A]	After expiry of MEAs [B]		
Galsulfase	Refund	1,665.2	1,308.3	10.3	21.4%
Eculizumab	Refund	6,451.0	4,498.1	7.0	30.3%
Lenalidomide	Refund	212.7	166.6	4.0	21.7%
Crizotinib	Refund	108.7	46.8	4.0	57.0%
Pirfenidone	Refund	5.0	3.0	2.1	40.8%
Clofarabine	CED	1,744.1	1,744.1	5.0	N/A
	Average			5.4	34.2%

## Discussion

Since 2014, the Korean government has introduced MEAs to facilitate access to new premium priced medicines under the NHI’s pharmaceutical benefit scheme. This study explored problems, policy, and political backgrounds of MEA introduction, based on Kingdon’s policy process, and analysed a total of 48 medicines listed through MEA contracts from January 2014 to December 2020, as well as the subsequent consequences of MEAs in South Korea.

In particular, the MEA has been systematized and consolidated under Moon Care, the mantra of “listing non-listable medicines.” Through MEAs—irrespective of whether they are combined with other bypass mechanisms—a substantial number of new medicines for cancer or orphan diseases have been listed, with a shorter review process and increased likelihood of listing. This is consistently confirmed by previous studies (Yoo et al., 2019). The MEAs were found to improve patient’s access to new high-cost anti-cancer medicines in South Korea (Kim et al., 2017; Kim et al., 2020). Additionally, Gong et al. (2020) found that the odds of the positive listing recommendations for new medicines was higher (OR = 1.53 95% CI 1.01–2.33) when comparing the situations before and after the MEA implementation.

We have shown that in Korea, finance-based contracts have been preferred over performance-based contracts, which was consistent with other countries where finance-based MEAs have accounted for the majority of schemes (Ferrario et al., 2017; Bouvy et al., 2018; Rick et al., 2020; Zampirolli Dias et al., 2020). However, our findings revealed significant differences in the selection of MEA types by countries. For example, Italy has actively adopted performance-based MEAs (50%), while Korea employed only 2.1%. In the same context, it was reported that only outcome-based types such as CED and CTC were predominantly used in the Netherlands, Sweden, and Italy (Kanavos et al., 2017; Bouvy et al., 2018; Frisk et al., 2018; IQVIA, 2019). For example, Eculizumab indicated for paroxysmal nocturnal haemoglobinuria has been contracted with the CED in Sweden and Netherlands, while in Korea, it was contracted under the refund scheme. Outcome-based risk-sharing contracts have an advantage in improving the efficiency of resource allocation by helping solve uncertainty about the health outcomes of new medicines and producing evidence based on the real world (Carlson et al., 2017), while other things such as data collection, setting endpoints for outcome measures and the subject of performance evaluation need to be addressed with stakeholders beforehand (Klemp et al., 2011). This is because there are considerable challenges to be addressed for an increase in outcome-based schemes. Key challenges include the ability of the healthcare system to collect pertinent patient-level data in routine clinical practice, who owns the data, issues of privacy surrounding patient-level data, instigating such schemes early potentially gives support for new medicines with still very limited data, and will the company pay back the resources spent on the new medicine if it fails to achieve the desired outcomes in routine clinical care (Zampirolli Dias et al., 2020; Godman et al., 2021). The latter was seen with the drug Olaratumab, resulting in substantial losses in some European countries and regions (Pontes et al., 2020). Concerns with the extent of meaningful patient-level data that can be collected in routine clinical care, have resulted in the national health system in Scotland instigating the Cancer Medicines Outcomes Programme (CMOP) to test the feasibility of routinely collecting and analysing pertinent patient reported outcome measures (PROMs) (Baillie et al., 2020; MacBride-Stewart et al., 2021). Despite these concerns, outcome-based contracts have the potential to efficiently prevent financial burden due to uncertainty and pursue appropriateness in utilization by developing rational grounds for their implementation and follow-up (Carlson et al., 2010).

Furthermore, we found that MEAs have become the norm (instead of being the exception) for new cancer medicines or those for orphan diseases in Korea. Previous studies recommended MEAs be an exceptional pathway and not a norm for listing in the Korean NHI, but this has now changed (Klemp et al., 2011; Department of Health DoH, 2014; Christiane and Soumana, 2018; Moorkens et al., 2021).

We are aware that there have been concerns regarding MEAs, in addition to those discussed with regard to the outcome-based scheme (Zampirolli Dias et al., 2020; Godman et al., 2021). A study analysing drug expenditures from 2014 to 2018 emphasized that the average annual growth rates for medicines for cancer and rare diseases were 15.4% and 21.6%, respectively, indicating that they contributed significantly to escalating drug expenditures in Korea during this period (Luzzatto et al., 2018). Yoo et al. (2019) found that the growth rate of drug expenditures was 14.9% from 2015 (12,389 million USD) to 2017 (14,244 million USD), while those for medicines under MEAs was 51.5% (from 91 million USD to 228.8 million USD). Kim et al. (2020) conducted a price comparison between medicines with MEA contracts and those undergoing the standard HTA process; they found that new medicines with MEAs tended to be priced two times higher than the comparators. Consequently, MEAs—even though they provide earlier access to high-priced medicines in cancer or rare diseases—do have a negative impact on the budget financing of the NHI, which needs to be taken into consideration because most new medicines being developed are for cancer and rare diseases, with typical high price expectations (Luzzatto et al., 2018; Godman et al., 2021; IQVIA, 2022).

In conclusion, MEAs have been introduced in Korea as an alternative to address patient’s access to anticancer agents or orphan drugs, coupling with policy and politics streams that intended to list all non-listedable services including pharmaceuticals. MEAs have been actively used to circumvent rigorous HTA process due to the nature of less effective but costly medicines.

Our study confirmed that MEAs have played a critical role in ensuring access to medicines for cancer or rare diseases since their introduction in Korea. However, the listing of medicines whose cost-effectiveness is uncertain has increased, and follow-up measures are insufficient in terms of effectiveness and budgetary impact of these medicines. Therefore, MEAs are still incompatible with the principle of “value for money” and challenge the sustainable budget impact and transparency of Korean pharmaceutical benefit policy.

This study has some limitations. As the real prices of medicines under MEAs were not disclosed, we could not measure the impact of MEAs accurately. The confidentiality that is a part of MEAs impedes the transparency of policy process, which is the intrinsic goal of public policy (Carlson et al., 2010; Ferrario and Kanavos, 2013; Department of Health DoH, 2014; Ferrario et al., 2017; Sabine and Kenneth, 2017; Christiane and Soumana, 2018; Luzzatto et al., 2018; Park, 2018; Subramaniam et al., 2021). In particular, information regarding the benefits and risks associated with the MEA contracts should be clearly disseminated to all key stakeholders. However, further information to evaluate MEAs could not be found; this situation is similar to other countries, where access to information on MEAs has been limited. Nevertheless, we believe this study was the first attempt to evaluate South Korea’s MEAs *via* Kingdon’s policy model and analysed the policy impacts in light of the listing and pricing of medicines, compared to foreign experiences.

## Conclusion

Since 2014, MEAs have been implemented in South Korea to address the issue of access to medicines for cancers or rare diseases. Despite concerns about the MEA, it has been systematized and consolidated under Moon care with the mantra of “listing all non-listed services.” Consequently, a substantial number of new medicines for cancer or orphan diseases have been listed with a shorter review process and increased likelihood of listing. Although they were introduced as a bypass mechanism, MEAs have now superseded the standard process for these medicines. Further studies are needed to evaluate the impact of the confidential agreements in light of the issue of access to medicines, and uncertainties regarding financial burdens and the effectiveness of new high-priced medicines with limited clinical data at launch.

## Data Availability

The original contributions presented in the study are included in the article/Supplementary Material, further inquiries can be directed to the corresponding authors.
