# Supplementary material for: Introduction of managed entry agreements in Korea: Problem, policy, and politics
Source: Front Pharmacol. 2023 Apr 13;14:999220. doi: 10.3389/fphar.2023.999220 (PMC10133550; doi:10.3389/fphar.2023.999220)
Supplement: Supplementary file 1 [file Table1.docx]

Appendix: Medicines contracted with MEAs in South Korea

| No | Brand name | Active ingredient | Manufacturer | Major indication | Type of the MEA | Applied year | Current status |
| --- | --- | --- | --- | --- | --- | --- | --- |
| 1 | Naglazyme* | galsulfase | Samoh | mucopolysaccharidosis | Refund | 2014 | Expired at the manufacturer’s request |
| 2 | Soliris* | eculizumab | Handok | nocturnal hemoglobinuria | Refund | 2014 | Expired at the manufacturer’s request |
| 3 | Evoltra* | clofarabine | Sanofi | Leukemia | CED | 2014 | Expired due to the proved effectiveness |
| 4 | Erbitux | cetuximab | Merck | colorectal carcinoma | Refund | 2014 | Valid |
| 5 | Revlimid | lenalidomide | Celgene | multiple myeloma | Refund | 2014 | Expired due to generic entry |
| 6 | Xtandi | enzalutamide | Astellas | prostate cancer | Refund | 2014 | Valid |
| 7 | Xalkori | crizotinib | Pfizer | lung cancer | Refund | 2015 | Expired due to alternative drug listing |
| 8 | Pirespa | pirfenidone | Ildong | idiopathic pulmonary fibrosis | Refund | 2015 | Expired due to generic entry |
| 9 | Caprelsa | vandetanib | Sanofi | thyroid cancer | Expenditure cap | 2015 | Valid |
| 10 | Stivarga | regorafenib | Bayer | gastrointestinal stromal tumour | Refund | 2016 | Valid |
| 11 | Imbruvica | ibrutinib | Janssen | mantle cell lymphoma | Expenditure cap | 2016 | Valid |
| 12 | Vimizim | elosulfase alfa | Samoh | mucopolysaccharidosis | Expenditure cap | 2016 | Valid |
| 13 | Blincyto | blinatumomab | Amgen | Leukemia | Expenditure cap | 2016 | Valid |
| 14 | Diterin | sapropterin | Alvogen | phenylketonuria | Expenditure cap | 2017 | Valid |
| 15 | Pomalyst | pomalidomide | Celgene | multiple myeloma | Refund | 2017 | Valid |
| 16 | Defitelio | defibrotide | Handok | veno-occlusive disease | Expenditure cap | 2017 | Valid |
| 17 | Perjeta | pertuzumab | Roche | breast cancer | Utilization cap | 2017 | Valid |
| 18 | Zelboraf | vemurafenib | Roche | melanoma | Expenditure cap | 2017 | Valid |
| 19 | Kadcyla | trastuzumab emtansine | Roche | breast cancer | Utilization cap | 2017 | Valid |
| 20 | Opdivo | nivolumab | Ono | lung cancer | Refund mix** | 2017 | Valid |
| 21 | Keytruda | pembrolizumab | MSD | lung cancer | Refund mix** | 2017 | Valid |
| 22 | Tafinlar | dabrafenib | Novartis | melanoma | Expenditure cap | 2017 | Valid |
| 23 | Lynparza | olaparib | Astrazeneca | ovarian cancer | Expenditure cap | 2017 | Valid |
| 24 | Alecensa | alectinib | Roche | lung cancer | Expenditure cap | 2017 | Valid |
| 25 | Mekinist | trametinib | Novartis | melanoma | Expenditure cap | 2017 | Valid |
| 26 | Ibrance | palbociclib | Pfizer | breast cancer | Refund | 2017 | Valid |
| 27 | Olita | olmutinib | Hanmi | lung cancer | Expenditure cap | 2017 | Valid |
| 28 | Tagrisso | osimertinib | Astrazeneca | lung cancer | Refund | 2017 | Valid |
| 29 | Tecentriq | atezolizumab | Roche | urothelial carcinoma | Expenditure cap | 2018 | Valid |
| 30 | Sylvant | siltuximab | Janssen | castlemanbe capinom | Expenditure cap | 2018 | Valid |
| 31 | Kyprolis | carfilzomib | Amgen | multiple myeloma | Refund | 2018 | Valid |
| 32 | Iclusig | ponatinib | Otsuka | Leukemia | Expenditure cap | 2018 | Valid |
| 33 | Cyramza | ramucirumab | Lilly | gastric cancer | Refund | 2018 | Valid |
| 34 | Vyndaqel | tafamidis meglumine | Pfizer | polyneuropathy | Expenditure cap | 2018 | Valid |
| 35 | Praxbind | idarucizumab | Boehringer | coagulant | Expenditure cap | 2019 | Valid |
| 36 | Cabometyx | cabozantinib | Ipsen | renal cell carcinoma | Utilization cap | 2019 | Valid |
| 37 | Darzalex | daratumumab | Janssen | multiple myeloma | Refund mix** | 2019 | Valid |
| 38 | Spinraza | nusinersen | Biogen | spinal amyotrophy | Refund | 2019 | Valid |
| 39 | Besponsa | inotuzumab | Pfizer | Leukemia | Expenditure cap | 2019 | Valid |
| 40 | Zejula | niraparib | Takeda | ovarian cancer | Expenditure cap | 2019 | Valid |
| 41 | Dupixent | dupilumab | Sanofi | atopic dermatitis | Refund | 2020 | Valid |
| 42 | Imfinzi | durvalumab | Astrazeneca | lung cancer | Refund | 2020 | Valid |
| 43 | Venclexta | venetoclax | Abbvie | Leukemia | Expenditure cap | 2020 | Valid |
| 44 | Hemlibra | emicizumab | JW | hemophilia | Expenditure cap | 2020 | Valid |
| 45 | Strensiq | asfotase | Handok | hypophosphatasia | Refund mix** | 2020 | Valid |
| 46 | Verzenio | abemaciclib | Lilly | breast cancer | Refund | 2020 | Valid |
| 47 | Bavencio | avelumab | Merck | Merkel cell carcinoma | Refund | 2020 | Valid |
| 48 | Kisqali | ribociclib | Novartis | breast cancer | Refund | 2020 | Valid |

* These medicines were contracted as a pilot plan of refund scheme before the MEA was introduced.

** Refund mix is the agreement that combines refund type and other type of the MEA.
